# Comparison of Reporting and Transparency in Published Protocols and Publications in Umbrella Reviews: Scoping Review

**DOI:** 10.2196/43299

**Published:** 2023-08-02

**Authors:** Liang Zhao, Caiyi Shen, Ming Liu, Jiaoyan Zhang, Luying Cheng, Yuanyuan Li, Lanbin Yuan, Junhua Zhang, Jinhui Tian

**Affiliations:** 1 Evidence-Based Medicine Center School of Basic Medical Sciences Lanzhou University Lanzhou China; 2 The First Clinical Medical College Lanzhou University Lanzhou China; 3 Evidence-Based Nursing Center School of Nursing Lanzhou University Lanzhou China; 4 Zigong First People's Hospital Zigong China; 5 Evidence-Based Medicine Center Tianjin University of Traditional Chinese Medicine Tianjin China

**Keywords:** umbrella reviews, protocol, publication, inconsistency, transparency

## Abstract

**Background:**

Inconsistencies between a protocol and its umbrella review (UR) may mislead readers about the importance of findings or lead to false-positive results. Furthermore, not documenting and explaining inconsistencies in the UR could reduce its transparency. To our knowledge, no study has examined the methodological consistency of the protocols with their URs and assessed the transparency of the URs when generating evidence.

**Objective:**

This study aimed to investigate the inconsistency of protocols with their URs in the methodology and assess the transparency of the URs.

**Methods:**

We searched medical-related electronic databases from their inception to January 1, 2022. We investigated inconsistencies between protocols and their publications and transparencies in the search strategy, inclusion criteria, methods of screening and data extraction, quality assessment, and statistical analysis.

**Results:**

We included 31 protocols and 35 publications. For the search strategy, 39 inconsistencies between the protocols and their publications were found in 26 of the 35 (74%) URs, and 16 of these inconsistencies were indicated and explained. There were 84 inconsistencies between the protocols and their URs regarding the inclusion criteria in 31 of the 35 (89%) URs, and 29 of the inconsistencies were indicated and explained. Deviations from their protocols were found in 12 of the 32 (38%) URs reporting the methods of screening, 14 of the 30 (47%) URs reporting the methods of data extraction, and 11 of the 32 (34%) URs reporting the methods for quality assessment. Of the 35 URs, 6 (17%) were inconsistent with their protocols in terms of the tools for quality assessment; one-half (3/6, 50%) of them indicated and explained the deviations. As for the statistical analysis, 31 of the 35 (89%) URs generated 61 inconsistencies between the publications and their protocols, and 16 inconsistencies were indicated and explained.

**Conclusions:**

There was a high prevalence of inconsistencies between protocols and publications of URs, and more than one-half of the inconsistencies were not indicated and explained in the publications. Therefore, how to promote the transparency of URs will be a major part of future work.

## Introduction

Umbrella reviews (URs) are clusters that encompass previously published systematic reviews or meta-analyses that consider many comparisons of interventions for the management of the same disease or condition, to provide an overall examination [1]. URs not only offer the possibility to address a broad scope of issues related to a topic of interest but are also ideal to highlight where the evidence base for a question is consistent or if contradictory findings exist and to explore and detail the reasons why [2]. In recent years, URs have become increasingly influential in biomedical literature, and they represent one of the highest levels of evidence synthesis currently available [3,4].

However, since a broad synthesis of data from many systematic reviews requires a high level of subject matter expertise and methodological skills, not all published URs follow clearly described, standardized methodology, which may reduce the credibility of evidence from URs [2,4-6]. Published protocols could leverage the strength of peer review to help authors refine their study design [7]. Furthermore, they allow the tracking of inconsistencies between the protocol and its publication, and disclosed and explained inconsistencies make it possible for readers to evaluate potential bias [8-10].

Inconsistencies may exist because of conscious or subconscious manipulation to reach the desired conclusion, which misleads readers about the importance of findings or causes false-positive results [11-13]. In some cases, valid reasons may exist for modifying a protocol in the course of undertaking a study. However, deviations from the protocol are often poorly documented and explained, and this makes it impossible to tell whether the changes introduce bias, thus reducing the transparency of the evidence [14,15]. Unfortunately, inconsistencies between protocols and their publications are common in randomized controlled trials, systematic reviews, and core outcomes set in the medical field, and most inconsistencies are not disclosed and discussed in the publications, making their transparency very worrying [14-16]. Previous studies that have assessed the reporting and methodological quality of URs have suggested that the transparency of URs is deficient [17]. However, to our knowledge, no study has examined the methodological consistency of a protocol with its UR and assessed its transparency by determining whether inconsistencies were documented and explained in the UR.

The primary aim of this study was to investigate inconsistencies between a protocol and its publication when developing an UR. The secondary aim was to assess the transparency of the URs when generating evidence.

## Methods

### Eligibility Criteria

URs that met the following criteria were included: (1) A UR was defined as a quantitative or qualitative synthesis of medical-related systematic reviews, (2) a UR protocol and its publication along with their methodological descriptions were available, (3) URs’ protocols and their publications were published in peer-reviewed journals or preprints, (4) the UR was part of a study, (5) the UR was published in English. There was no restriction on the time frame of the studies. We excluded the following studies: (1) methodological reviews of URs, scoping or rapid reviews, qualitative reviews, integrative reviews, and evidence synthesis and (2) abstracts, conference proceedings, and letters to editors.

### Search Strategy

A comprehensive literature search was performed in 7 medical-related databases, including PubMed, Embase, Web of Science, The Cochrane Library, Joanna Briggs Institute (JBI) Database of Systematic Reviews and Implementation Reports, PROSPERO, and Open Science Framework (OSF), from the inception of these databases to January 1, 2022. The search strategies were developed by an expert in literature searches (JT), and the full search strategies are presented in Table S1 in Multimedia Appendix 1. We also manually searched the reference lists of included studies for potentially eligible studies.

### Study Selection

We managed all records using EndNote (version X9, Clarivate Analytics) software and removed duplicates using automatic and manual screening. The study selection was divided into 4 steps. First, 2 authors (LZ and ML) independently screened all URs’ protocols and publications using title, abstract, and full text, consecutively. Second, we read the full text of identified publications to find the protocols mentioned in the articles (JZ and LC). Third, for protocols that were not related to included publications in the previous step, the study names or standard abbreviations (where applicable) combined with the term “umbrella” were used to search PubMed, Embase, The Cochrane Library, and Web of Science for their publications (YL, CS, and LY). In cases where the same protocol was described in multiple publications, all related publications were included. Finally, the reference lists of included URs were manually searched for additional studies. Any disagreements were resolved through discussion between authors and, if necessary, consultation with senior authors (JZ and JT).

### Data Extraction

We developed the data extraction form and revised it after piloting it on a random sample of 20% of included URs (LZ, CS, and ML). The extracted data consisted of 2 parts: general characteristics and methodological characteristics. A list of extracted items is given in Table S2 in Multimedia Appendix 1. For any observed inconsistencies between a protocol and its publication, we investigated whether the inconsistencies had been disclosed and explained in the publications and then extracted the text details of the explanation. Of the 5 authors, 2 authors (LZ and JZ) independently extracted data from the protocols and their publications, and another 3 authors (ML, CS, and LC) checked the extracted data. Conflicts were resolved by discussion between authors and, if necessary, consultation with senior authors (JZ and JT).

### Inconsistency and Transparency Assessment

According to the JBI manual for URs [2], we assessed inconsistencies between protocols and their publications in 6 areas: search strategy, inclusion criteria, methods for screening, methods for data extraction, methodological quality assessment, and statistical analysis. For each area, the text details where an inconsistency existed were extracted from both the protocol and its publication. Inconsistency was defined as follows: any deviation between a protocol and its publication that altered the substance or meaning of an area or fully reported in the protocol or UR but only briefly or not reported in the UR or protocol [18,19]. The definition of consistency was the substance or meaning regarding the compared area was identical in the protocol and its publication, so changes in style, wording, tense, or abbreviations were not considered [19]. The inconsistency assessment in each area was based on an internal standard that was developed, independently pilot-tested (in 5 pairs), and revised by 2 authors (LZ and ML; Table S3 in Multimedia Appendix 1). Transparency was defined as deviations from a protocol that were disclosed and explained in its UR [15].

### Statistical Analysis

The frequency of inconsistencies and transparency were our primary outcomes. Median (IQR) was used for continuous variables, including the number of authors, journal impact factor, total number of studies included, and interval between the protocol and its publication, whereas frequency (%) was used for categorical variables. The statistical analyses were conducted with SPSS Statistics v26.0 (IBM Corp).

## Results

### Search and Selection Results

**Figure 1:**
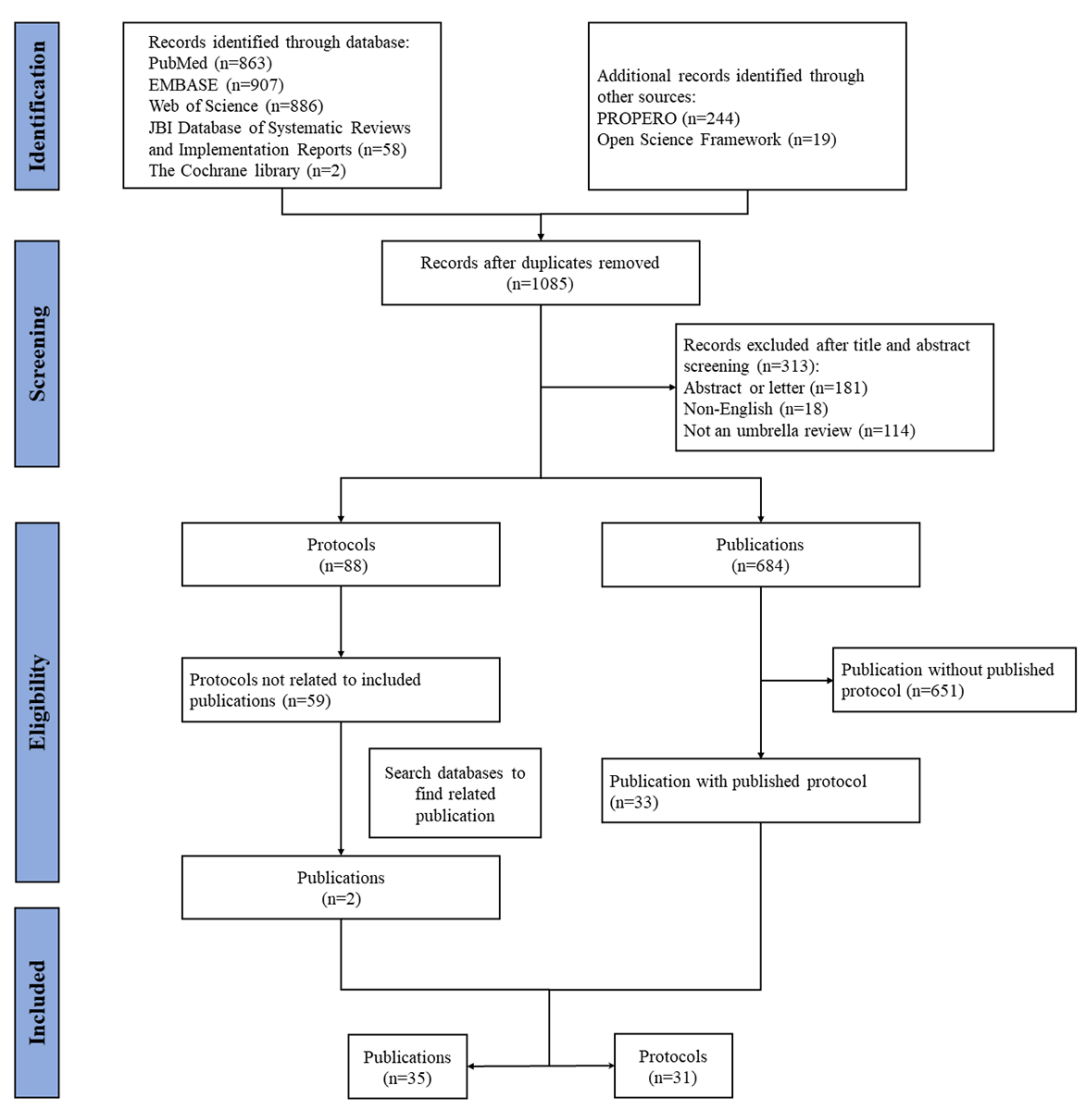
The flowchart of the screening process.

The database search yielded 2716 records, and an additional 263 records were identified through other sources. Following the removal of duplicates and title and abstract screening, 88 protocols and 684 publications were screened using the full text. In total, we identified 31 protocols and 35 publications (Figure 1). The full list of included URs can be found in Multimedia Appendix 2.

### Characteristics of Included URs

The included URs were published between 2012 and 2021, and most of them (60/66, 91%) were published after 2016 (Multimedia Appendix 3). The interval between the protocols and their publications ranged from 1 month to 5 years and had a median interval of 2 (IQR 1-2) years. These URs were conducted in 14 countries, with Australia ranking first in both protocols and publications (Multimedia Appendix 4). Of the protocols, 30 were published in 4 journals with a median journal impact factor of 3.007 (IQR 0.000-3.136), whereas 34 publications were published in 27 journals with a median journal impact factor of 3.312 (IQR 2.329-5.926). The median number of authors of the protocols (median 6, IQR 4-8 authors) was the same as the publications (median 6, IQR 5-9 authors). Regarding registrations, 23 URs were registered in PROSPERO or OSF. Compared with the protocols, more publications indicated sources of financial support (23 vs 30) and conflicts of interests (2 vs 4). The number of reviews included in URs ranged from 1 to 114, with a median of 14 (IQR 7-36). The characteristics of the included URs are summarized in Table 1, Table S4 in Multimedia Appendix 5, and Multimedia Appendix 6.

**Table 1:** The characteristics of the included umbrella reviews’ protocols and publications.

Characteristics		Protocols (n=31)	Publications (n=35)
Journal impact factor^a^, median (IQR)		3.007 (0.000-3.136)	3.312 (2.329-5.926)
**Journal impact factor, n (%)**			
	0.0 to 3.0	1 (3)	6 (17)
	>3.0 to 6.0	20 (65)	14 (40)
	>6.0	0 (0)	8 (23)
	Non-SCI^b^	9 (29)	6 (17)
	Preprint	1 (3)	1 (3)
Number of authors, median (IQR)		6 (4-8)	6 (5-9)
**Number of authors**, **n (**%**)**			
	2-5	13 (42)	13 (37)
	6-10	15 (48)	17 (49)
	>10	3 (10)	5 (14)
**Registration, n (%)**			
	PROSPERO	21 (68)	23 (66)
	Open Science Framework	1 (3)	1 (3)
	Not registered	9 (29)	11 (31)
**Funding, n (%)**			
	Yes	18 (58)	26 (74)
	No	5 (16)	4 (11)
	Not reported	8 (26)	5 (14)
**Conflicts of interest, n (%)**			
	Yes	2 (6)	4 (11)
	No	29 (94)	31 (89)

^a^The journal impact factor was determined using the 2021 Journal Citation Report.

^b^SCI: Science Citation Index.

### Inconsistencies and Transparency in the Search Strategy

All URs described the search strategy, and 39 inconsistencies between the protocols and their publications were found in 26 of the 35 URs (74%). Of 39 inconsistencies, 16 (41%) were indicated and explained in the publications (Table 2, Table S5 in Multimedia Appendix 5, and Multimedia Appendix 6).

**Table 2:** The frequency and transparency of inconsistencies in 6 fields in the 35 publications included in the umbrella reviews (URs).

Items	Publications in the URs reporting each item, n	Frequency, n (%)	Transparency, n (%)
Search strategy	35	26 (74)	16 (41)
Literature sources	35	21 (60)	3 (14)
Language restrictions	34	5 (15)	4 (80)
Search time	35	13 (37)	9 (69)
Inclusion criteria	35	31 (89)	29 (35)
Participants	35	13 (37)	5 (38)
Interventions	35	16 (46)	6 (38)
Comparators	26	10 (38)	4 (40)
Outcomes	35	19 (54)	7 (37)
Type of studies	35	13 (37)	2 (15)
Other inclusion criteria	24	13 (54)	5 (38)
Methods for screening	32	12 (38)	2 (17)
Methods for data extraction	30	14 (47)	3 (21)
Quality assessment	35	13 (37)	6 (35)
Methods for quality assessment	32	11 (34)	3 (27)
Tools for quality assessment	35	6 (17)	3 (50)
Statistical analysis	35	31 (89)	16 (26)
Overlap	18	3 (17)	0 (0)
Certainty of evidence	24	13 (54)	2 (15)
Summary of findings	26	12 (46)	3 (25)
Data analysis	35	9 (26)	4 (44)
Effect size	13	11 (85)	2 (18)
Other statistical analysis	21	14 (67)	5 (36)

Of the 35 publications, 21 (60%) deviated from their protocols in the literature sources: 14 deviations occurred in the databases, and 12 URs generated deviations regarding other literature sources. Of the 21 URs, 3 (14%) indicated inconsistencies in the publications. Compared with the protocols, 1 publication expanded the range of language for included studies, and the other added language restrictions to the included studies. Although 3 publications did not describe language restrictions, their protocols did. In the 5 URs, 4 (80%) inconsistencies were disclosed in the URs. Regarding search times, 13 of the 35 (37%) publications differed from their protocols: 6 because of updates, 3 for other reasons, and the remaining 4 lacked an explanation.

### Inconsistencies and Transparency in Inclusion Criteria

All URs described the inclusion criteria, and 31 of the 35 (89%) URs had inconsistencies between the protocols and their publications (Table 2, Table S6 in Multimedia Appendix 5, and Multimedia Appendix 6).

For the participants, 13 of the 35 (37%) publications were inconsistent with their protocols. In the 13 URs, 5 described the participants in detail in the protocols but not in the publications. Compared with the protocols, 6 narrowed the inclusion of the participants, and 2 expanded the inclusion of the participants. Of the 13 publications, 5 (38%) indicated and explained the inconsistencies.

Of the 35 URs, 16 (46%) generated the following inconsistencies between the protocols and their publications in the interventions: (1) 6 publications narrowed the scope of the interventions compared with the protocols; (2) compared with the protocols, the scope of the interventions was extended in 4 publications; (3) 3 publications were contradictory to the protocols; (4) 3 publications did not describe the interventions, while their protocols did. Of the 16 publications, 6 (38%) pointed out and interpreted the inconsistencies.

Regarding the comparators, 10 of the 26 (38%) URs showed deviations between the protocols and their publications: 4 publications increased the number of comparators compared with their protocols, 4 protocols but not their publications described the comparators, and 2 publications removed comparators that were included in the protocols. Of these 10 URs, 4 (40%) publications indicated where the inconsistencies arose.

Inconsistencies between the protocols and their publications in the outcomes were found in 19 of the 35 (54%) URs. The most common inconsistency was that the publications reduced the number of outcomes compared with their protocols (8 URs). The second was that the publications added outcomes (4 URs). The third was that the protocols but not the publications described the outcomes (4 URs). Finally, 3 publications changed the outcomes described in the protocols. Inconsistencies were indicated in 7 of the 19 (37%) publications.

Inconsistencies between the protocols and their publications regarding the type of studies were found in 13 of the 35 (37%) URs: (1) 6 publications narrowed the inclusion of the study design compared with their protocols; (2) 5 publications expanded the inclusion of the study design, and 1 of them added inclusion of updated randomized controlled trials; (3) 2 protocols but not their publications described the type of included studies. The reasons for deviations were explained by 2 of the 13 (15%) URs.

Other inclusion criteria were reported by 24 of the 35 (69%) URs, of which 14 (58%) URs reported the requirements for included studies, 11 (46%) URs reported the quality of included studies, and only 2 (8%) URs reported the requirements of the protocol. In the other inclusion criteria, there were the following inconsistencies between the protocols and their publications in 13 of the 24 (54%) URs: 8 inconsistencies in the requirements for included studies, 5 inconsistencies in the quality of included studies, and 2 inconsistencies in the requirements of the protocol. The inconsistencies were detailed in 5 of the 13 (38%) publications.

### Inconsistencies and Transparency in Screening, Quality Assessment, and Data Extraction

The following inconsistencies between the protocols and their publications were found in the methods for screening, data extraction, and quality assessment (Table 2, Table S7 in Multimedia Appendix 5, and Multimedia Appendix 6): (1) 14 inconsistencies were identified when resolving disagreements among reviewers; (2) 8 URs did not describe the methods, while their protocols did; (3) 8 inconsistencies were found in the number of reviewers; (4) 7 publications described the methods, while the protocols did not. The publications described 8 of the inconsistencies.

All URs described tools for quality assessment; the most common (13/35, 37%) was the JBI Critical Appraisal Checklist for Systematic Reviews and Research Syntheses. The tools used in the protocols were changed in 3 publications, and 3 publications did not depict tools, while their protocols did. The deviations were indicated in 3 of the 6 (50%) publications.

### Inconsistencies and Transparency in Statistical Analyses

We found 62 inconsistencies between the protocols and their publications in 31 of the 35 (89%) URs, and 16 of the 62 (26%) inconsistencies were indicated and explained in the publications (Table 2, Table S8 in Multimedia Appendix 5, and Multimedia Appendix 6).

Of the 35 URs, 18 described how to deal with the overlaps of primary studies between included systematic reviews. There were inconsistencies between 3 of the 18 (17%) protocols and their publications. Of them, 2 protocols gave ways to deal with overlaps, while their publications did not implement them; 1 publication added the calculation of the corrected covered area. None of the inconsistencies was indicated in the publications.

Tools for assessing the certainty of the evidence were reported by 24 URs, and 20 of these URs used the Grading of Recommendations Assessment, Development, and Evaluation (GRADE) criteria. Inconsistencies existed between 13 of the 24 (54%) URs and their protocols: 10 protocols but not their publications reported assessments of the certainty of the evidence, and 3 publications added tools for assessing the credibility of evidence that were not described in the protocols. Inconsistencies were detailed by 2 of the 13 (15%) URs.

What findings to present and how to present them were described in 26 URs: 12 of the 26 (46%) URs generated inconsistencies between the protocols and their publications, and 3 URs indicated and explained inconsistencies in the publications. Compared with the protocols, inconsistencies could be categorized as follows: (1) 5 publications differed on whether to apply the “summary of evidence” table, (2) the forms of evidence presentation were changed in 5 publications, and (3) 2 publications changed the reported elements.

All URs depicted the data analysis, and inconsistencies between the protocols and their publications were found in 9 of the 35 (26%) URs. Compared with the protocols, 3 publications removed quantitative analysis, 2 publications added quantitative analysis, 2 publications altered the rules for qualitative synthesis, 1 publication changed the quantitative analysis model, and 1 protocol did not describe the data analysis while the publication did. Of the 9 inconsistencies, 4 (44%) were detailed and explained in the publications.

How to standardize the effect sizes was reported in 13 URs, and 11 of these 13 (85%) URs had inconsistencies between the protocols and their publications: 8 URs only reported it in the publications, and 1 UR only reported it in the protocol; regarding the standardization of dichotomous data, 2 publications were inconsistent with their protocols. Of the 11 inconsistencies, 2 (18%) were indicated and explained in the publications.

Of the 35 URs, 21 described other statistical analyses, including subgroup analysis (17/35, 49%), sensitivity analysis (6/35, 17%), and publication bias or small study effects (6/35, 17%). Inconsistencies between the protocols and their publications were found in 14 of the 21 (67%) URs. Regarding subgroup analyses, 12 URs had inconsistencies with their protocols. Of the 12 URs, 9 only reported the subgroup analysis in the protocols, 2 only reported it in the publications, and 1 publication changed the subgroup analyses that were described in the protocol. Deviations between the protocols and their publications in publication bias or small study effects and sensitivity analysis were found in 4 URs and 1 UR, respectively. Of the 14 URs with inconsistencies, 5 (36%) detailed the inconsistencies in the publications.

## Discussion

We identified 31 protocols and 35 publications of the URs. Inconsistencies between the protocols and their publications were found in all areas, with inconsistencies occurring most frequently in inclusion criteria and statistical analyses. In addition, less than one-half of the inconsistencies were indicated in the publications of URs, and the transparency of URs was inadequate.

### Inconsistencies in Methodology

Our study showed inconsistencies in the search strategy between the protocols and their publications in 74% (26/35) of the URs, which was similar to previous result that indicated 77% of reviews changed the search strategy between the protocols and their publications [18]. Changes in the database and other literature sources between the protocols and their publications were frequent in the URs. This significantly affects the completeness and comprehensiveness of the included studies [20]. Although it has been recommended that biomedical citation databases complemented by other literature sources is the best combination for identifying comprehensive reviews, there was no guidance on which specific databases should be searched when conducting URs [21-23]. Thus, the specific combination of literature sources that could identify more available evidence should be clarified in the future. To avoid missing relevant citations, it seems like a beneficial change for the publication to expand the range of languages for included studies compared with the protocol [24]. However, we only found this change in 1 UR [25]. In addition, we also found quite a few inconsistencies in the search time frame, but it was acceptable that the main reasons were to update or avoid outdated literature.

Although URs often aim to provide a broad overview of the evidence on a topic, it is still important to be specific about the inclusion criteria [26]. These criteria provide not only a guide for the readers to clearly understand what is proposed by the authors but also a guide for the reviewers themselves to make decisions about the studies to be included in the URs [2]. More importantly, changes in inclusion criteria were much less likely to be detected as a potential source of bias [27]. In this study, 89% (31/35) of the URs had inconsistencies between their protocols and publications in the inclusion criteria, with the highest volume of inconsistencies in the outcomes, which was higher than the prevalence of inconsistencies in outcomes among the protocols and their publications of systematic reviews in a study [16] that included reviews published between 2002 and 2009. Modifications (that is, addition, removal, or reprioritization) of outcomes that have been based on prior knowledge of the results might introduce the possibility of bias into the URs, mislead clinical decision-makers, and possibly jeopardize medical safety, and adherence to the published protocol can be effective in avoiding potential bias [13,28]. Noteworthy, after the publication of the PRISMA (Preferred Reporting Items for Systematic Reviews and Meta-Analyses) guidelines in 2009 [29], the prevalence of inconsistencies in outcomes was reduced in another study, which restricted it to reviews published between 2011 and 2014 [30]. However, a validated systematic reporting standard that specializes in URs is not available, which needs to be further developed.

In terms of the statistical analysis, we identified that 89% (31/35) of the URs’ publications deviated from their protocols. This is similar to a finding for systematic reviews [18], in which 89% of publications were inconsistent with their protocols in the statistical analysis. One-quarter of the publications changed the data analysis described in the protocols. These changes might be a source of complexity and bias in the analysis and, worse still, lead to a significant variation in the conclusions [26,31]. Because of heterogeneity between the reviews, it might not always be feasible to conduct a quantitative synthesis in URs [32,33]. However, a narrative synthesis could become complex and open to bias if not adequately described, and there was a concern that synthesis errors at the review level could result in errors at the UR level [31]. Therefore, appropriate data analysis in URs needs to be further explored.

Over one-half of the included URs dealt with overlaps of primary studies, which was consistent with the findings by Pieper et al [34], and 12 URs did not report it in the publications. This could potentially lead to the statistical power being overestimated and thus risk producing a misleading, overly precise estimate [2,35,36]. Thus, how to deal with overlapping of primary studies in the URs should be strengthened. Although only one-third of the URs converted the results presented across the systematic reviews to one common summary statistic, 73% (8/11) of inconsistencies were shown by the fact that the differences were reported in the publications but not in the protocols. This seems like a beneficial change, as a common effect size could make the comparison more straightforward [4]. In addition, we found that the most common inconsistency between the protocol and their publication in the subgroup analysis, sensitivity analysis, and publication bias was their removal from publications. This would weaken the level of evidence of an effect and the richness of the picture of evidence [4]. These methods were not mentioned in the URs not only in our study but also in most of the guidelines [31]. Therefore, it is necessary to pay more attention to the subgroup analysis, sensitivity analysis, and publication bias in URs.

As for certainty of evidence, the most common inconsistency was that the GRADE was removed in the publications, which might lead to a diminished certainty of evidence generated by URs [2,26]. Although most of the guidelines recommended that the GRADE be used to appraise the certainty of evidence, how to apply the GRADE in URs was not yet available [31]. In addition, some criteria in the GRADE were only applicable to the level of primary studies when assessing the body of evidence, so it was inappropriate to be directly transferred to URs [37,38]. Therefore, a modification to the GRADE so it could also be applied to URs would be beneficial and practical, and many authors are already familiar with it. Furthermore, some URs [39-41] were found that added approaches to assess the certainty of evidence in the publications; it is also acceptable to include additional approaches to assess the certainty of evidence if they are objective and standardized.

### Inadequate Transparency

Research transparency can speed scientific progress, can increase trust in science, and is one of the core values of science [42,43]. In this study, the transparency was inadequate; it fluctuated between 17% and 41% across the 6 areas, which was similar to the findings of previous studies [15,18]. The majority of inconsistencies between the protocols and their publications were not indicated and explained in the publications, and this made it difficult to provide definitive conclusions as to why these changes may occur and if they introduced potential bias. The inadequate transparency of URs might have the cumulative effect of producing a distorted body of evidence with too few null effects and many false positives, exaggerating the effectiveness of programs; it could also threaten medical safety [44,45]. Therefore, how to promote the transparency of URs will be a major part of future work.

Publishing a protocol in a peer-reviewed journal is a crucial way to improve transparency, by comparing the protocol with a completed UR to detect whether unintended and undocumented changes were made [13]. However, in our study, the protocols were published in only 4 journals, while the publications were published in 27 journals. This demonstrates that the number of peer-reviewed journals that published the URs’ protocols was very limited, and few protocols were published. Therefore, it is necessary to encourage more journals to accept protocols for publication. Furthermore, editors or peer reviewers could also compare publications in the URs with their protocols and check back to confirm whether the changes were indicated and explained to promote transparency.

A study published in the journal *Science* pointed out that systematic reporting standards also contribute to the transparency of research [42]. For example, PRISMA 2020 requires authors to describe and explain any amendments to the information provided in the protocol [46]. It provides authors with guidance to report and explain any inconsistencies. However, a validated UR-specific systematic reporting standard has not yet emerged and needs to be developed in the future. Furthermore, the current reward structure of publication fails to encourage transparent studies, which was evidenced by the greater likelihood of publication of statistically significant, novel, and theoretically tidy results than null, replicated, or perplexing results, even at the expense of transparency of research [42,44]. Therefore, greater awareness is needed that null results are as important as statistically significant results in helping others to more accurately assess the evidence base for a program.

### Strengths and Limitations

To the best of our knowledge, our study was the first study to assess inconsistent reporting between the protocols and the publications of URs in the methodology. Because we comprehensively analyzed each step of the development of an UR, our findings could be considered beneficial to the production of URs. However, there were some limitations in our study. First, only URs published in English were included. Second, our study conducted only an exploratory analysis with a relatively small sample size, so some inconsistencies may be overlooked. Third, some publications depict only part of the methodology of an UR, resulting in incomplete comparisons between protocols and publications. Last, there is no standard protocol to address the analysis of inconsistencies between protocols and their publications; therefore, we created our protocol and analysis process based on previous similar studies. There is potential for bias or that other factors were not included.

### Implications for Future Research and Practice

There are several areas for future development in the methodology of URs, including (1) the specific combination of literature sources that could identify more available evidence, (2) development of a validated reporting quality tool specifically for URs, (3) appropriate and adequate use of statistical analysis in URs, and (4) modification to the GRADE so that it can be applied in URs. It is also beneficial if our study can help medical editors and scientific journals draw attention to whether a UR’s protocol exists and identify any unrecognized or unreasonable changes between the protocols and their publications during the editing process.

### Conclusions

There was a high prevalence of inconsistencies between protocols and publications of URs, especially in the inclusion criteria and statistical analysis. More worryingly, more than one-half of URs did not indicate and explain the inconsistencies, and the transparency was inadequate. Thus, the authors of URs should be required to describe and explain any deviation from their protocols. Our study provides further evidence that published protocols allow the tracking of any changes that have taken place, to assess the transparency of URs. Therefore, authors of URs are encouraged to publish their protocols, and journals are urged to accept protocols for publication. In addition, editors or peer reviewers could compare publications of URs with their protocols and check back to confirm whether the changes were indicated and explained.

## Appendix

Multimedia Appendix 1 Search strategy and extracted information.

Multimedia Appendix 2 List of all included protocols and publications.

Multimedia Appendix 3 Published years of the included umbrella reviews (URs)’ protocols and publications (inner circle represents the UR protocol; outer circle represents the corresponding publication for the protocol; the length of the square is proportional to the year of publication).

Multimedia Appendix 4 Countries of corresponding authors of the included URs’ protocols and publications.

Multimedia Appendix 5 Information regarding the inconsistencies in the characteristics of publications compared with protocols.

Multimedia Appendix 6 The specifics of inconsistencies between the protocol and its publication for each UR.

## Abbreviations

GRADE: Grading of Recommendations Assessment, Development, and Evaluation
JBI: Joanna Briggs Institute
OSF: Open Science Framework
PRISMA: Preferred Reporting Items for Systematic Reviews and Meta-Analyses
URs: umbrella reviews
