# Supplementary material for: Comparison of Reporting and Transparency in Published Protocols and Publications in Umbrella Reviews: Scoping Review
Source: J Med Internet Res. 2023 Aug 2;25:e43299. doi: 10.2196/43299 (PMC10433027; doi:10.2196/43299)
Supplement: Multimedia Appendix 1 [file jmir_v25i1e43299_app1.docx]

**Table S1** The search strategy.

| **Database** | **Search strategy** | **Records** |
| --- | --- | --- |
| PubMed (2022.01.01) | "Umbrella Review"[Title/Abstract] OR "Umbrella Reviews"[Title/Abstract] OR "umbrella systematic review"[Title/Abstract] OR "umbrella systematic reviews"[Title/Abstract] OR "umbrella meta-analysis"[Title/Abstract] OR "umbrella synthesis"[Title/Abstract] OR "umbrella systematic literature review"[Title/Abstract] OR "umbrella systematic literature reviews"[Title/Abstract] | 863 |
| Embase (2022.01.01) | "Umbrella Review":ti,ab,kw OR "Umbrella Reviews":ti,ab,kw OR "umbrella systematic review":ti,ab,kw OR "umbrella systematic reviews":ti,ab,kw OR "umbrella meta-analysis":ti,ab,kw OR "umbrella synthesis":ti,ab,kw OR "umbrella systematic literature review":ti,ab,kw OR "umbrella systematic literature reviews":ti,ab,kw | 907 |
| Web of Science (2022.01.01) | TS=("Umbrella Review" OR "Umbrella Reviews" OR "umbrella systematic review" OR "umbrella systematic reviews" OR "umbrella meta-analysis" OR "umbrella synthesis" OR "umbrella systematic literature review" OR "umbrella systematic literature reviews") | 886 |
| JBI Database of Systematic Reviews and Implementation Reports (2022.01.01) | #1 Title: "Umbrella Review" OR "Umbrella Reviews" OR "umbrella systematic review" OR "umbrella systematic reviews" OR "umbrella meta-analysis" OR "umbrella synthesis" OR "umbrella systematic literature review" OR "umbrella systematic literature reviews"  #2 Abstract: "Umbrella Review" OR "Umbrella Reviews" OR "umbrella systematic review" OR "umbrella systematic reviews" OR "umbrella meta-analysis" OR "umbrella synthesis" OR "umbrella systematic literature review" OR "umbrella systematic literature reviews"  #3 #1 OR #2 | 58 |
| The Cochrane library (2022.01.01) | "Umbrella Review":ti,ab,kw OR "Umbrella Reviews":ti,ab,kw OR "umbrella systematic review":ti,ab,kw OR "umbrella systematic reviews":ti,ab,kw OR "umbrella meta-analysis":ti,ab,kw OR "umbrella synthesis":ti,ab,kw OR "umbrella systematic literature review":ti,ab,kw OR "umbrella systematic literature reviews":ti,ab,kw | 2 |

**Table S2** A list of extracted items.

| General characteristics | Methodological characteristics | |
| --- | --- | --- |
| Year of publications | Search strategy | Literature sources |
| Number of authors |  | Language restrictions |
| Country of the corresponding author |  | Search time |
| Journal | Inclusion criteria | Participants |
| 2021 Journal Impact Factor^a^ |  | Interventions |
| Registration |  | Comparators |
| Funding (yes, no, or not report) |  | Outcomes |
| Conflicts of interests (yes, no) |  | Type of studies |
| Total number of studies included |  | Other inclusion criteria |
| Interval between protocol and its publication | Methods for screening |  |
|  | Methods for data extraction |  |
|  | Methodological quality assessment | Tools for methodological quality assessment |
|  |  | Methods for methodological quality assessment |
|  | Statistical analysis | How to deal with overlap |
|  |  | Tools for assessing the credibility of evidence |
|  |  | Summary of finding, i.e. what findings to present and how to present them |
|  |  | Data analysis, i.e. qualitative, quantitative or semiquantitative synthesis |
|  |  | How to standardize the effect size |
|  |  | Other statistical analysis |

a, Journal impact factor was provided by the 2021 Journal Citation Report.

**Table S3** An internal standard used to assess the inconsistency in each area.

| Area | Compared component | Explanation | Example |
| --- | --- | --- | --- |
| Search strategy | Literature sources | The number or name of databases or other sources were inconsistent between the protocol and its publication; Fully reported in the protocol/UR, but only briefly or not reported in the UR/protocol. | A database or other source (e.g., a list of references to retrieved articles) were added in the publication; The literature sources was reported in both protocol and publication, but it was modified in the publication (from the DARE database to the Cochrane database); An UR described the literature sources in detail in the protocols but didn’t in the publications. |
|  | Language restrictions | Language restriction on the inclusion of studies was inconsistent between the protocol and its publication; Fully reported in the protocol/UR, but only briefly or not reported in the UR/protocol. | The protocol included English language SRs but its publication indicated no language restriction; The protocol included English language SRs but its publication not reported language restriction. |
|  | Search time | The search start time or search cut-off time in the protocol does not match its publications; Fully reported in the protocol/UR, but only briefly or not reported in the UR/protocol. | Database searches was restricted to reviews published during or after 2008 in the protocol, but its publication searched from July 2009; The protocol reported the search time frame but its publications not reported. |
| Inclusion criteria | Participants | The scope of the included populations was inconsistent between the protocol and its publication; Fully reported in the protocol/UR, but only briefly or not reported in the UR/protocol. | The age limitation of participants was reported in the protocol but was removed in its publication; URs described the participants in detail in the protocols but didn’t in the publications. |
|  | Interventions | The scope of the included interventions was inconsistent between the protocol and its publication; Fully reported in the protocol/UR, but only briefly or not reported in the UR/protocol. | The diet and exercise as an intervention was reported in the protocol but was removed in its publication; The publication added decompressive craniectomy as an intervention; Publications didn’t describe the interventions while their protocols did. |
|  | Comparators | The scope of the included comparators was inconsistent between the protocol and its publication; Fully reported in the protocol/UR, but only briefly or not reported in the UR/protocol. | The behavioral intervention as a comparator was reported in the protocol but was removed in its publication; The publication added placebo as a comparator; An UR described comparators in the protocol but not in the publication. |
|  | Outcomes | The scope of the included outcomes was inconsistent between the protocol and its publication; Fully reported in the protocol/UR, but only briefly or not reported in the UR/protocol. | The outcomes were reported in both protocol and publication, but it was modified in the publication (from false negative rate to intracranial injury); The publication added quality of life as an outcome; An UR described outcomes in the protocol but not in the publication. |
|  | Type of studies | The scope of included study design was inconsistent between the protocol and its publication; Fully reported in the protocol/UR, but only briefly or not reported in the UR/protocol. | The study design was reported in both protocol and publication, but it was modified in the publication (from observational studies to experimental study design); The UR supplemented with RCTs in the publication; One protocol described the type of studies but not in the publication. |
|  | Other inclusion criteria | The scope of other inclusion criteria (such as reporting requirements, quality of studies) was inconsistent between the protocol and its publication; Fully reported in the protocol/UR, but only briefly or not reported in the UR/protocol. | The publication included high-quality SRs, but the protocol does not specify; The reporting requirements of included SRs was reported in both protocol and publication, but it was modified in the publication; An UR required to clarify the existence of the protocols in the publication but not in the protocol. |
| Methods for screening | | The number of reviewers, how they screened or how disagreements were resolved were inconsistent between the protocol and its publication; Fully reported in the protocol/UR, but only briefly or not reported in the UR/protocol. | The method for screening was reported in both protocol and publication, but it was modified in the publication (such as from pairs of four to pairs of three, from one reviewer screened and checked by a second reviewer to two reviewers independently); The publication added the description of resolving disagreements among reviewers; Publications of URs didn’t describe methods, but their protocols did. |
| Methods for data extraction | | The number of reviewers, how they extracted or how disagreements were resolved were inconsistent between the protocol and its publication; Fully reported in the protocol/UR, but only briefly or not reported in the UR/protocol. | The method for data extraction was reported in both protocol and publication, but it was modified in the publication (such as from pairs of four to pairs of three, from one reviewer extracted and checked by a second reviewer to two reviewers independently); The publication added the description of resolving disagreements among reviewers; Publications of URs didn’t describe methods, but their protocols did. |
| Methodological quality assessment | Tools for methodological quality assessment | The tool for methodological quality assessment was inconsistent between the protocol and its publication; Fully reported in the protocol/UR, but only briefly or not reported in the UR/protocol. | The tool was reported in both protocol and publication, but it was modified in the publication (from AMSTAR to AMSTAR 2); Publications of URs didn’t describe the tool, but their protocols did. |
|  | Methods for methodological quality assessment | The number of reviewers, how they assessed or how disagreements were resolved were inconsistent between the protocol and its publication; Fully reported in the protocol/UR, but only briefly or not reported in the UR/protocol. | The method for quality assessment was reported in both protocol and publication, but it was modified in the publication (such as from pairs of four to pairs of three, from one reviewer assessed and checked by a second reviewer to two reviewers independently); The publication added the description of resolving disagreements among reviewers; Publications of URs didn’t describe methods, but their protocols did. |
| Statistical analysis | How to deal with overlap | How to deal with overlap of original research studies in the included systematic reviews was inconsistent between the protocol and its publication; Fully reported in the protocol/UR, but only briefly or not reported in the UR/protocol. | The way to deal with overlap was reported in both protocol and publication, but it was modified in the publication (from any overlap was clearly indicated to the corrected covered area was calculated); Publications of URs didn’t describe the way to deal with overlap, but their protocols did. |
|  | Tools for assessing the credibility of evidence | The tool for assessing the credibility of evidence was inconsistent between the protocol and its publication; Fully reported in the protocol/UR, but only briefly or not reported in the UR/protocol. | Publications added the tool for assessing the credibility of evidence that didn’t describe in the protocols; Publications of URs didn’t describe the way to deal with overlap, but their protocols did. |
|  | Summary of finding | What findings to present and how to present them was inconsistent between the protocol and its publication; Fully reported in the protocol/UR, but only briefly or not reported in the UR/protocol. | URs increased or decreased the “Summary of evidence” table in their publications; The publications added reported heterogeneity, risk of bias; Publications of URs didn’t describe the summary of finding, but their protocols did. |
|  | Data analysis | Data analysis (i.e. qualitative, quantitative or semiquantitative synthesis) was inconsistent between the protocol and its publication; Fully reported in the protocol/UR, but only briefly or not reported in the UR/protocol. | The publication replenished the quantitative analysis; The data analysis was reported in both protocol and publication, but it was modified in the publication (from generalized linear mixed models to bayesian Markov-chain Monte Carlo method); One protocol didn’t depict the data analysis, but the publication did |
|  | Effect size | The way to standardize effect size was inconsistent between the protocol and its publication; Fully reported in the protocol/UR, but only briefly or not reported in the UR/protocol. | The way to standardize effect size was reported in both protocol and publication, but it was modified in the publication (from ORs to SMDs); Publications of URs didn’t describe the way to standardize effect size, but their protocols did. |
|  | Other statistical analysis | Other statistical analysis (such as subgroup analysis, sensitivity analysis, and publication bias or small-study effects) was inconsistent between the protocol and its publication; Fully reported in the protocol/UR, but only briefly or not reported in the UR/protocol. | The methods for examining publication bias was reported in both protocol and publication, but it was modified in the publication (from Egger’s test to the Doi plot and the Luis Furuya-Kanamori index); Publications of URs didn’t describe subgroup analysis, but their protocols did. |
