# Supplementary material for: Comparison of Reporting and Transparency in Published Protocols and Publications in Umbrella Reviews: Scoping Review
Source: J Med Internet Res. 2023 Aug 2;25:e43299. doi: 10.2196/43299 (PMC10433027; doi:10.2196/43299)
Supplement: Multimedia Appendix 2 [file jmir_v25i1e43299_app2.docx]

**Appendix file 2 List of all included protocols and publications.**

| **Protocols** | **Publications** |
| --- | --- |
| Damery S, Flanagan S, Combes G. The effectiveness of interventions to achieve co-ordinated multidisciplinary care and reduce hospital use for people with chronic diseases: study protocol for a systematic review of reviews. Syst Rev 2015; 4:64. | Damery S, Flanagan S, Combes G. Does integrated care reduce hospital activity for patients with chronic diseases? An umbrella review of systematic reviews. BMJ Open 2016;6(11):e011952. |
| Elliott J, Kelly SE, Bai Z, et al. Optimal duration of dual antiplatelet therapy following percutaneous coronary intervention: protocol for an umbrella review. BMJ Open 2017;7(4):e015421. | Elliott J, Kelly SE, Bai Z, et al. Optimal Duration of Dual Antiplatelet Therapy Following Percutaneous Coronary Intervention: An Umbrella Review. Can J Cardiol 2019;35(8):1039-1046. |
| Campbell JM, Peters MD. The association of chemotherapy-induced toxicities with germline polymorphisms: an umbrella review of systematic reviews and meta-analyses. JBI Database System Rev Implement Rep 2014;12(10):40-46. | Campbell JM, Bateman E, Stephenson MD, et al. Methotrexate-induced toxicity pharmacogenetics: an umbrella review of systematic reviews and meta-analyses. Cancer Chemother Pharmacol 2016;78(1):27-39. |
|  | Campbell JM, Bateman E, Peters MD, et al. Fluoropyrimidine and platinum toxicity pharmacogenetics: an umbrella review of systematic reviews and meta-analyses. Pharmacogenomics 2016;17(4):435-451. |
|  | Campbell JM, Stephenson MD, Bateman E, et al. Irinotecan-induced toxicity pharmacogenetics: an umbrella review of systematic reviews and meta-analyses. Pharmacogenomics J 2017;17(1):21-28. |
| Chai L K, Burrows T, May C, et al. Effectiveness of family-based weight management interventions in childhood obesity: an umbrella review protocol. JBI Database System Rev Implement Rep 2016;14(9):32-39. | Chai L K, Collins C, May C, et al. Effectiveness of family-based weight management interventions for children with overweight and obesity: an umbrella review. JBI database of systematic reviews and implementation reports 2019;17(7): 1341-1427. |
| Jadczak AD, Makwana N, Luscombe-Marsh ND, et al. Effectiveness of exercise interventions on physical function in community-dwelling frail older people: An umbrella review protocol. JBI Database System Rev Implement Rep 2016;14(9):93-102. | Jadczak AD, Makwana N, Luscombe-Marsh N, et al. Effectiveness of exercise interventions on physical function in community-dwelling frail older people: An umbrella review of systematic reviews. JBI Database System Rev Implement Rep 2018;16(3):752-775. |
| Qin D, Yue L, Xue B, et al. Pharmacological treatments for patients with irritable bowel syndrome: An umbrella review of systematic reviews and meta-analyses. Medicine (Baltimore) 2019;98(32):e15920. | Chen M, Tang TC, Qin D, et al. Pharmacologic Treatments for Irritable Bowel Syndrome: an Umbrella Systematic Review. J Gastrointestin Liver Dis 2020;29(2):199-209. |
| Tardif PA, Moore L, Lauzier F, et al. Low-value clinical practices in adult traumatic brain injury: an umbrella review protocol. BMJ Open 2019;9(10):e031747. | Moore L, Tardif PA, Lauzier F, et al. Low-Value Clinical Practices in Adult Traumatic Brain Injury: An Umbrella Review. J Neurotrauma 2020;37(24):2605-2615. |
| Dinsdale S, Azevedo LB, Shucksmith J, et al. Effectiveness of weight management, smoking cessation and alcohol reduction interventions in changing behaviors during pregnancy: an umbrella review protocol. JBI Database System Rev Implement Rep 2016;14(10):29-47. | Heslehurst N, Hayes L, Jones D, et al. The effectiveness of smoking cessation, alcohol reduction, diet and physical activity interventions in changing behaviours during pregnancy: A systematic review of systematic reviews. PLoS One 2020;15(5):e0232774. |
| Schultz TJ, Roupas P, Wiechula R, et al. Nutritional interventions for optimizing healthy body composition in older adults in the community: A protocol for an umbrella review of studies of effectiveness and qualitative perceptions and experiences. JBI Database System Rev Implement Rep 2014;12(11):31-47 | Schultz TJ, Roupas P, Wiechula R, et al. Nutritional interventions for optimizing healthy body composition in older adults in the community: An umbrella review of systematic reviews. JBI Database System Rev Implement Rep 2016;14(8):257-308. |
| Apóstolo J, Cooke R, Bobrowicz-Campos E, et al. Predicting risk and outcomes for frail older adults: a protocol for an umbrella review of available frailty screening tools. JBI Database System Rev Implement Rep 2016;13(12):14-24. | Apóstolo J, Cooke R, Bobrowicz-Campos E, et al. Predicting risk and outcomes for frail older adults: an umbrella review of frailty screening tools. JBI Database System Rev Implement Rep 2017;15(4):1154-1208. |
| Goldstein KM, McDuffie JR, Shepherd-Banigan M, et al. Nonpharmacologic, nonherbal management of menopause-associated vasomotor symptoms: an umbrella systematic review (protocol). Syst Rev 2016;5:56. | Goldstein KM, Shepherd-Banigan M, Coeytaux RR, et al. Use of mindfulness, meditation and relaxation to treat vasomotor symptoms. Climacteric 2017;20(2):178-182. |
|  | Shepherd-Banigan M, Goldstein KM, Coeytaux RR, et al. Improving vasomotor symptoms; psychological symptoms; and health-related quality of life in peri- or post-menopausal women through yoga: An umbrella systematic review and meta-analysis. Complement Ther Med. 2017;34:156-164. |
|  | Befus D, Coeytaux RR, Goldstein KM, et al. Management of Menopause Symptoms with Acupuncture: An Umbrella Systematic Review and Meta-Analysis. J Altern Complement Med 2018;24(4):314-323. |
| Thomson K, Bambra C, McNamara C, et al. The effects of public health policies on population health and health inequalities in European welfare states: protocol for an umbrella review. Syst Rev 2016;5:57. | Thomson K, Hillier-Brown F, Todd A, et al. The effects of public health policies on health inequalities in high-income countries: an umbrella review. BMC Public Health 2018;18(1):869. |
| Naik Y, Baker P, Walker I, et al. The macro-economic determinants of health and health inequalities-umbrella review protocol. Syst Rev 2017;6(1):222. | Naik Y, Baker P, Ismail SA, et al. Going upstream - an umbrella review of the macroeconomic determinants of health and health inequalities. BMC Public Health 2019;19(1):1678. |
| Melka AS, Chojenta CL, Holliday EG, et al. Effectiveness of pharmacotherapy for smoking cessation: protocol for umbrella review and quality assessment of systematic reviews. Syst Rev 2018;7(1):210. | Melka AS, Chojenta CL, Holliday EG, et al. Effectiveness of pharmacotherapy for smoking cessation: Umbrella review and quality assessment of systematic reviews. Research Square[Preprint]. July 30, 2020[cited 2022 Jan 22]. Available from: <https://doi.org/10.21203/rs.2.18915/v3> |
| Petrovskaya O, Lau F, Antonio M. Synthesising evidence on patient portals: a protocol for an umbrella review. BMJ Open 2019;9(3):e024469. | Antonio MG, Petrovskaya O, Lau F. The State of Evidence in Patient Portals: Umbrella Review. J Med Internet Res 2020;22(11):e23851. |
| Santos E, Duarte C, Marques A, et al. Effectiveness of non-pharmacological and non-surgical interventions on the impact of rheumatoid arthritis: an umbrella review protocol. JBI Database System Rev Implement Rep 2019;17(1):22-27. | Santos EJF, Duarte C, Marques A, et al. Effectiveness of non-pharmacological and non-surgical interventions for rheumatoid arthritis: an umbrella review. JBI Database System Rev Implement Rep 2019;17(7):1494-1531. |
| Skelton K, Herbert A, Benjamin-Neelon SE. Garden-based interventions and early childhood health: a protocol for an umbrella review. Syst Rev 2019;8(1):310. | Skelton KR, Lowe C, Zaltz DA, et al. Garden-based interventions and early childhood health: an umbrella review. Int J Behav Nutr Phys Act 2020;17(1):121. |
| Zhao Q, Shen J, Lu J, et al. Clinical efficacy, safety and tolerability of aliskiren monotherapy: a protocol for an umbrella review. BMJ Open. 2020;10(1):e033448. | Zhao Q, Shen J, Lu J, et al. Clinical efficacy, safety and tolerability of Aliskiren Monotherapy (AM): an umbrella review of systematic reviews. BMC Cardiovasc Disord 2020;20(1):179. |
| Lindekilde N, Scheuer S, Rutters F, et al. The prevalence of type 2 diabetes in people with psychiatric disorders: an umbrella review protocol. Syst Rev 2020;9(1):101. | Lindekilde N, Scheuer SH, Rutters F, et al. Prevalence of type 2 diabetes in psychiatric disorders: an umbrella review with meta-analysis of 245 observational studies from 32 systematic reviews. Diabetologia 2021;10.1007/s00125-021-05609-x. |
| Griswold DP, Gempeler A, Kolias AG, et al. Personal protective equipment for reducing the risk of COVID-19 infection among healthcare workers involved in emergency trauma surgery during the pandemic: an umbrella review protocol. BMJ Open 2021;11(3):e045598. | Griswold DP, Gempeler A, Kolias A, et al. Personal protective equipment for reducing the risk of COVID-19 infection among health care workers involved in emergency trauma surgery during the pandemic: An umbrella review. J Trauma Acute Care Surg 2021;90(4):e72-e80. |
| Hines S, Kynoch K, Khalil H. Effectiveness of interventions to prevent medication errors: an umbrella systematic review protocol. JBI Database System Rev Implement Rep 2018;16(2):291-296. | Khalil H, Kynoch K, Hines S. Interventions to ensure medication safety in acute care: an umbrella review. Int J Evid Based Healthc 2020;18(2):188-211. |
| Assi L, Rosman L, Chamseddine F, et al. Eye health and quality of life: an umbrella review protocol. BMJ Open 2020;10(8):e037648. | Assi L, Chamseddine F, Ibrahim P, et al. A Global Assessment of Eye Health and Quality of Life: A Systematic Review of Systematic Reviews. JAMA Ophthalmol 2021;139(5):526-541. |
| Marano L, Fusario D, Savelli V, et al. Robotic versus laparoscopic gastrectomy for gastric cancer: protocol for umbrella review of systematic reviews and meta-analyses. BMJ Open 2020;10(2):e033634. | Marano L, Fusario D, Savelli V, et al. Robotic versus laparoscopic gastrectomy for gastric cancer: an umbrella review of systematic reviews and meta-analyses. Updates Surg 2021;73(5):1673-1689. |
| van Esch BF, van der Zaag-Loonen HJ, Bruintjes TD, et al. Interventions for Menière's disease: protocol for an umbrella systematic review and a network meta-analysis. BMJ Open 2016;6(6):e010269. | van Esch BF, van der Zaag-Loonen H, Bruintjes T, et al. Interventions for Menière's disease: an umbrella systematic review. BMJ Evid Based Med 2021;bmjebm-2020-111410. |
| Besnier E, Thomson K, Stonkute D, et al. Which public health interventions are effective in reducing morbidity, mortality and health inequalities from infectious diseases amongst children in low-income and middle-income countries (LMICs): protocol for an umbrella review. BMJ Open 2019;9(12):e032981. | Besnier E, Thomson K, Stonkute D, et al. Which public health interventions are effective in reducing morbidity, mortality and health inequalities from infectious diseases amongst children in low- and middle-income countries (LMICs): An umbrella review. PLoS One 2021;16(6):e0251905. |
| Saygın Avşar T, McLeod H, Jackson L. Health outcomes of maternal smoking during pregnancy and postpartum period for the mother and infant: protocol for an umbrella review. Syst Rev 2018;7(1):235. | Avşar TS, McLeod H, Jackson L. Health outcomes of smoking during pregnancy and the postpartum period: an umbrella review. BMC Pregnancy Childbirth 2021;21(1):254. |
| O'Malley N, Clifford AM, Comber L, et al. Effectiveness of non-pharmacological falls prevention interventions for people with Multiple Sclerosis, Parkinson's Disease and stroke: protocol for an umbrella review. HRB Open Res 2020;3:17. | O'Malley N, Clifford AM, Conneely M, et al. Effectiveness of interventions to prevent falls for people with multiple sclerosis, Parkinson's disease and stroke: an umbrella review. BMC Neurol 2021;21(1):378. |
| Lindekilde N, Nefs G, Henriksen JE, et al. Psychiatric disorders as risk factors for the development of type 2 diabetes mellitus: an umbrella review protocol. BMJ Open 2019;9(5):e024981. | Lindekilde N, Rutters F, Erik Henriksen J, et al. Psychiatric disorders as risk factors for type 2 diabetes: An umbrella review of systematic reviews with and without meta-analyses. Diabetes Res Clin Pract 2021;176:108855. |
| Griebeler ML, Tsapas A, Brito JP, et al. Pharmacologic interventions for painful diabetic neuropathy: an umbrella systematic review and comparative effectiveness network meta-analysis (Protocol). Syst Rev 2012;1:61. | Griebeler ML, Morey-Vargas OL, Brito JP, et al. Pharmacologic interventions for painful diabetic neuropathy: An umbrella systematic review and comparative effectiveness network meta-analysis. Ann Intern Med 2014;161(9):639-649. |
| Alexandre K, Desrichard O, Burnand B, et al. Factors influencing self-management in adults with diabetes: an umbrella review protocol. JBI Database System Rev Implement Rep 2017;15(11):2630-2637. | Alexandre K, Campbell J, Bugnon M, et al. Factors influencing diabetes self-management in adults: an umbrella review of systematic reviews. JBI Evid Synth 2021;19(5):1003-1118. |
| Lugo A, Bosetti C, Peveri G, et al. Dose-response relationship between cigarette smoking and site-specific cancer risk: protocol for a systematic review with an original design combining umbrella and traditional reviews. BMJ Open 2017;7(10):e018930. | Liu X, Peveri G, Bosetti C, et al. Dose-response relationships between cigarette smoking and kidney cancer: A systematic review and meta-analysis. Crit Rev Oncol Hematol 2019;142:86-93. |
