# Supplementary figures and images for: Comparison of Reporting and Transparency in Published Protocols and Publications in Umbrella Reviews: Scoping Review
Source: J Med Internet Res. 2023 Aug 2;25:e43299. doi: 10.2196/43299 (PMC10433027; doi:10.2196/43299)

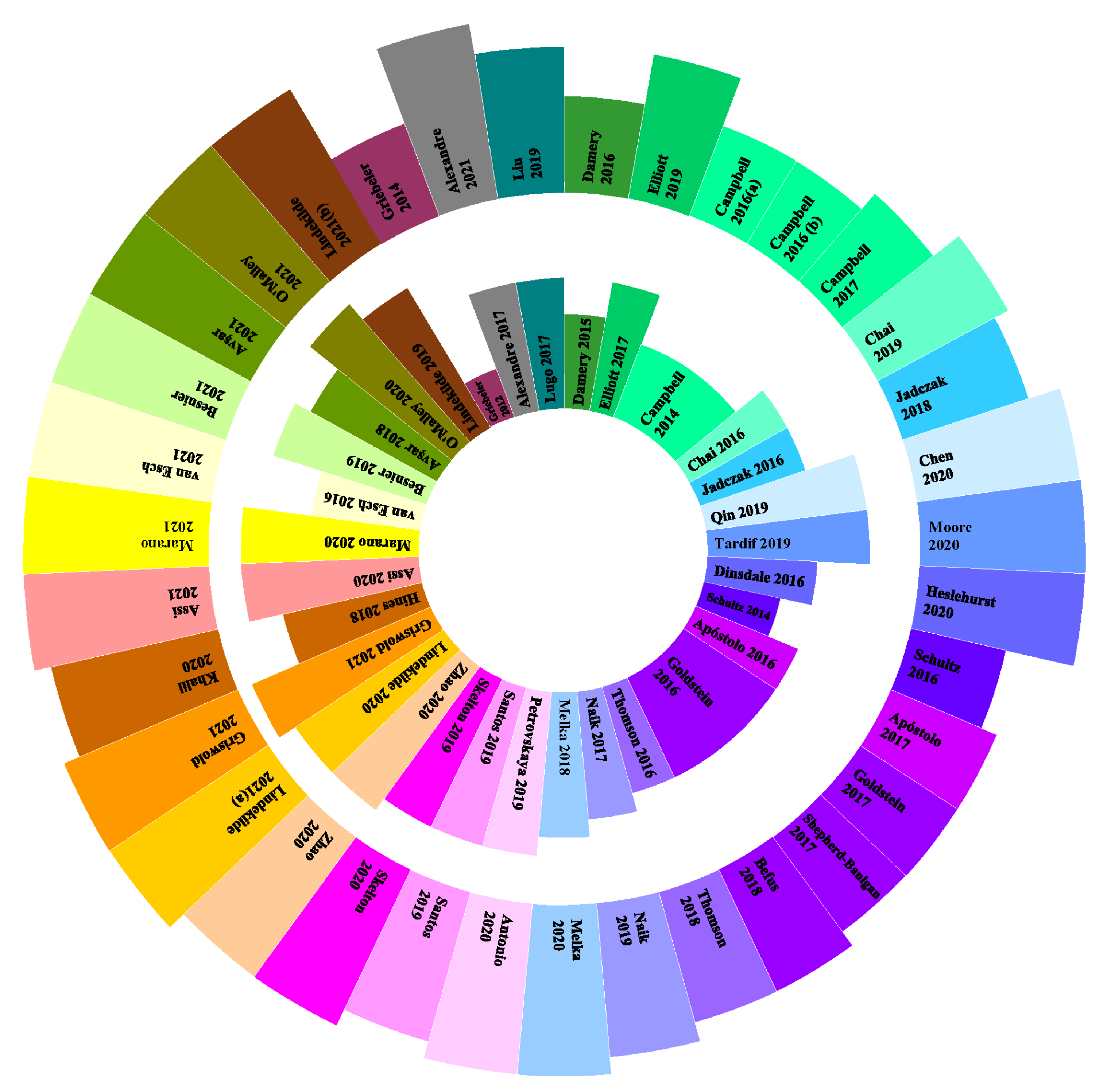

Supplement: Multimedia Appendix 3 [file jmir_v25i1e43299_app3.png]

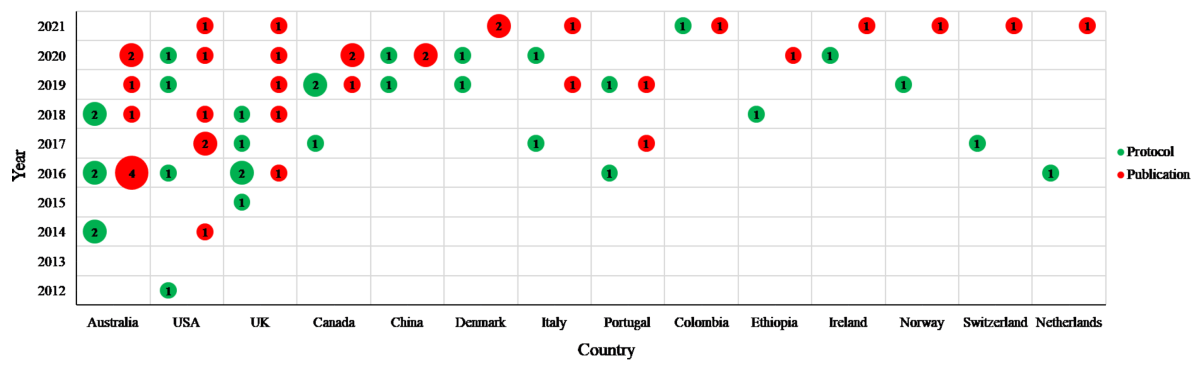

Supplement: Multimedia Appendix 4 [file jmir_v25i1e43299_app4.png]
