# Supplementary material for: Comparison of Reporting and Transparency in Published Protocols and Publications in Umbrella Reviews: Scoping Review
Source: J Med Internet Res. 2023 Aug 2;25:e43299. doi: 10.2196/43299 (PMC10433027; doi:10.2196/43299)
Supplement: Multimedia Appendix 5 [file jmir_v25i1e43299_app5.docx]

**Table S4.** Changes in publications in terms of general characteristics compared with protocols.

| Characteristics | Pairs of URs (n=35) |
| --- | --- |
| Journal impact factor^a^ |  |
| Increase | 16 (45.7%) |
| Same | 6 (17.1%) |
| Decrease | 6 (17.1%) |
| Non-SCI to SCI | 5 (14.3%) |
| Preprint to SCI | 1 (2.9%) |
| SCI to preprint | 1 (2.9%) |
| Number of authors |  |
| Increase | 18 (51.4%) |
| Same | 13 (37.1%) |
| Decrease | 4 (11.4%) |
| Registration |  |
| Same | 35 (97.1%) |
| Funding |  |
| Same | 27 (77.1%) |
| No funding to funding | 2 (5.7%) |
| Not report to funding | 4 (11.4%) |
| Not report to non-funding | 2 (5.7%) |
| Conflicts of interests |  |
| Same | 33 (94.3%) |
| Inconsistency | 2 (5.7%) |

a, Journal impact factor was provided by the 2021 Journal Citation Report. URs, umbrella reviews; SCI, science citation index.

**Table S5.** Details of inconsistencies in the search strategy of the included URs.

| Umbrella review | | Literature sources | | Language restrictions | | Search time | |  |
| --- | --- | --- | --- | --- | --- | --- | --- | --- |
| Protocol | Publication | Inconsistency | Transparency | Inconsistency | Transparency | Inconsistency | Transparency |  |
| Damery 2015 | Damery 2016 | Y | F | N | - | N | - |  |
| Elliott 2017 | Elliott 2019 | N | - | N | - | Y | T |  |
| Campbell 2014 | Campbell 2016 (a) | Y | F | N | - | Y | T |  |
|  | Campbell 2016 (b) | Y | F | N | - | Y | T |  |
|  | Campbell 2017 | Y | F | N | - | Y | T |  |
| Chai 2016 | Chai 2019 | Y | F | N | - | N | - |  |
| Jadczak 2016 | Jadczak 2018 | Y | F | N | - | N | - |  |
| Qin 2019 | Chen 2020 | Y | F | N | - | Y | F |  |
| Tardif 2019 | Moore 2020 | Y | F | N | - | N | - |  |
| Dinsdale 2016 | Heslehurst 2020 | Y | F | N | - | Y | F |  |
| Schultz 2014 | Schultz 2016 | N | - | N | - | N | - |  |
| Apóstolo 2016 | Apóstolo 2017 | N | - | N | - | N | - |  |
| Goldstein 2016 | Goldstein 2017 | Y | F | Y^a^ | T | N | - |  |
|  | Shepherd-Banigan 2017 | Y | F | N | - | N | - |  |
|  | Befus 2018 | Y | F | N | - | N | - |  |
| Thomson 2016 | Thomson 2018 | Y | F | N | - | N | - |  |
| Naik 2017 | Naik 2019 | Y | T | N | - | N | - |  |
| Melka 2018 | Melka 2020 | Y | F | N | - | Y | F |  |
| Petrovskaya 2019 | Antonio 2020 | Y | F | N | - | Y | T |  |
| Santos 2019 | Santos 2019 | N | - | N | - | N | - |  |
| Skelton 2019 | Skelton 2020 | N | - | NR  请在此放置您的文字 | - | N | - |  |
| Zhao 2020 | Zhao 2020 | N | - | N | - | N | - |  |
| Lindekilde 2020 | Lindekilde 2021(a) | N | - | N | - | N | - |  |
| Griswold 2021 | Griswold 2021 | N | - | N | - | Y | F |  |
| Hines 2018 | Khalil 2020 | N | - | N | - | N | - |  |
| Assi 2020 | Assi 2021 | Y | F | N | - | Y | T |  |
| Marano 2020 | Marano 2021 | N | - | Y^a^ | T | Y | T |  |
| van Esch 2016 | van Esch 2021 | Y | F | N | - | N | - |  |
| Besnier 2019 | Besnier 2021 | Y | T | N | - | Y | T | |
| Avşar 2018 | Avşar 2021 | N | - | N | - | N | - | |
| O'Malley 2020 | O'Malley 2021 | N | - | Y | T | N | - |  |
| Lindekilde 2019 | Lindekilde 2021(b) | N | - | N | - | N | - |  |
| Griebeler 2012 | Griebeler 2014 | N | - | N | - | Y | T |  |
| Alexandre 2017 | Alexandre 2021 | Y | T | Y | F | N | - |  |
| Lugo 2017 | Lin 2019 | Y | F | Y^a^ | T | N | - |  |

Abbreviations: Y, both the protocol and its UR described the methodology and the inconsistencies arose between them; Y^a^, the publication of UR did not describe the methodology, but the protocol did; T, the deviations from the protocol were indicated in its publication; F, the deviations from the protocol were not indicated in its publication; NR, neither the protocol nor its publication described the methodology.

**Table S6.** Details of inconsistencies in the inclusion criteria of the included URs.

| Umbrella review | | Participants | | | Interventions | | | Comparators | | Outcomes | | | Types of studies | | | | Other* | | |
| --- | --- | --- | --- | --- | --- | --- | --- | --- | --- | --- | --- | --- | --- | --- | --- | --- | --- | --- | --- |
| Protocol | Publication | Inconsistency | Transparency | Inconsistency | | Transparency | Inconsistency | | Transparency | | Inconsistency | Transparency | | Inconsistency | Transparency | Inconsistency | | Transparency |  |
| Damery 2015 | Damery 2016 | Y | F | Y | | F | N | | - | | Y | F | | Y | F | Y^b, 1^ | | T |  |
| Elliott 2017 | Elliott 2019 | Y^a^ | T | N | | - | N | | - | | Y | F | | N | - | Y^a, 2^ | | T |  |
| Campbell 2014 | Campbell 2016 (a) | N | - | Y | | F | NR | | - | | Y | F | | N | - | Y^b, 1^ | | F |  |
|  | Campbell 2016 (b) | N | - | N | | - | NR | | - | | Y | F | | Y | F | Y^b, 1^ | | F |  |
|  | Campbell 2017 | N | - | N | | - | NR | | - | | N | - | | Y | F | Y^b, 1^ | | F |  |
| Chai 2016 | Chai 2019 | Y | F | N | | - | N | | - | | N | - | | N | - | Y^b, 2, 3^ | | F |  |
| Jadczak 2016 | Jadczak 2018 | N | - | N | | - | N | | - | | N | - | | N | - | Y^2^ | | F |  |
| Qin 2019 | Chen 2020 | Y | F | Y | | F | N | | - | | Y | F | | N | - | NR | | - |  |
| Tardif 2019 | Moore 2020 | Y | F | Y | | F | Y | | F | | Y | F | | N | - | NR | | - |  |
| Dinsdale 2016 | Heslehurst 2020 | N | - | Y | | T | NR | | - | | Y | T | | Y | F | NR | | - |  |
| Schultz 2014 | Schultz 2016 | N | - | Y | | F | NR | | - | | Y | T | | N | - | Y^b, 2, 3^ | | F |  |
| Apóstolo 2016 | Apóstolo 2017 | Y | T | N | | - | N | | - | | N | - | | N | - | Y^b, 1^ | | T |  |
| Goldstein 2016 | Goldstein 2017 | N | - | Y | | F | N | | - | | Y | F | | Y | F | N^1^ | | - |  |
|  | Shepherd-Banigan 2017 | N | - | N | | - | N | | - | | N | - | | N | - | N^1^ | | - |  |
|  | Befus 2018 | N | - | Y | | F | N | | - | | N | - | | N | - | N^1^ | | - |  |
| Thomson 2016 | Thomson 2018 | N | - | Y | | F | N | | - | | Y | T | | Y | F | N^2^ | | - |  |
| Naik 2017 | Naik 2019 | N | - | Y | | T | N | | - | | N | - | | Y | F | NR | | - |  |
| Melka 2018 | Melka 2020 | N | - | N | | - | Y | | F | | N | - | | N | - | NR | | - |  |
| Petrovskaya 2019 | Antonio 2020 | N | - | N | | - | Y^a^ | | T | | Y^a^ | T | | Y | F | N^1, 2^ | | - |  |
| Santos 2019 | Santos 2019 | N | - | N | | - | Y | | F | | N | - | | N | - | N^1^ | | - |  |
| Skelton 2019 | Skelton 2020 | N | - | N | | - | NR | | - | | Y | F | | N | - | NR | | - |  |
| Zhao 2020 | Zhao 2020 | Y^a^ | F | N | | - | Y | | F | | Y | F | | N | - | NR | | - |  |
| Lindekilde 2020 | Lindekilde 2021(a) | N | - | N | | - | NR | | - | | N | - | | Y | F | Y^2^ | | F |  |
| Griswold 2021 | Griswold 2021 | N | - | Y | | T | N | | - | | Y | F | | Y | F | NR | | - |  |
| Hines 2018 | Khalil 2020 | Y | F | N | | - | NR | | - | | N | - | | N | - | Y^2^ | | F |  |
| Assi 2020 | Assi 2021 | Y | F | Y | | F | Y | | F | | Y | F | | N | - | N^2^ | | - |  |
| Marano 2020 | Marano 2021 | Y^a^ | T | Y^a^ | | T | Y^a^ | | T | | Y^a^ | T | | Y^a^ | T | Y^a, 2^ | | T |  |
| van Esch 2016 | van Esch 2021 | N | - | N | | - | N | | - | | N | - | | N | - | NR | | - |  |
| Besnier 2019 | Besnier 2021 | N | - | N | | - | Y | | F | | N | - | | N | - | N^2^ | | - |  |
| Avşar 2018 | Avşar 2021 | Y^a^ | T | Y^a^ | | T | Y^a^ | | T | | Y^a^ | T | | N | - | NR | | - |  |
| O'Malley 2020 | O'Malley 2021 | N | - | N | | - | N | | - | | Y | F | | N | - | N^1^ | | - |  |
| Lindekilde 2019 | Lindekilde 2021(b) | N | - | N | | - | N | | - | | N | - | | N | - | N^2^ | | - |  |
| Griebeler 2012 | Griebeler 2014 | Y | F | Y | | F | N | | - | | N | - | | Y | F | Y^a, 2^ | | T |  |
| Alexandre 2017 | Alexandre 2021 | N | - | N | | - | NR | | - | | N | - | | N | - | N^2^ | | - |  |
| Lugo 2017 | Lin 2019 | Y^a^ | T | Y^a^ | | T | Y^a^ | | T | | Y^a^ | T | | Y^a^ | T | NR | | - |  |

Abbreviations: Y, both the protocol and its UR described the methodology and the inconsistencies arose between them; Y^a^, the publication of UR did not describe the methodology, but the protocol did; Y^b^, the publication of UR described the methodology, but its protocol did not; T, the deviations from the protocol were indicated in its publication; F, the deviations from the protocol were not indicated in its publication; NR, neither the protocol nor its publication described the methodology; other*, including quality restrictions (1), reporting requirements (2), and the existence of a protocol (3).

**Table S7.** Details of inconsistencies in the methods for screening, data extraction, and quality assessment of the included URs.

| Umbrella review | | Methods for screening | | Methodological quality assessment | | | | Methods for data extraction | |
| --- | --- | --- | --- | --- | --- | --- | --- | --- | --- |
| Protocol | Publication | Inconsistency | Transparency | Methods | | Tools | | Inconsistency | Transparency |
|  |  |  |  | Inconsistency | Transparency | Inconsistency | Transparency |  |  |
| Damery 2015 | Damery 2016 | Y | F | Y | F | N | - | Y | F |
| Elliott 2017 | Elliott 2019 | N | - | Y^a^ | T | N | - | N | - |
| Campbell 2014 | Campbell 2016 (a) | NR | - | N | - | N | - | NR | - |
|  | Campbell 2016 (b) | NR | - | N | - | N | - | NR | - |
|  | Campbell 2017 | NR | - | N | - | N | - | NR | - |
| Chai 2016 | Chai 2019 | N | - | Y | F | N | - | NR | - |
| Jadczak 2016 | Jadczak 2018 | N | - | N | - | N | - | NR | - |
| Qin 2019 | Chen 2020 | Y | F | Y^b^ | F | Y | F | Y | F |
| Tardif 2019 | Moore 2020 | Y | F | N | - | N | - | Y | F |
| Dinsdale 2016 | Heslehurst 2020 | Y | F | Y | F | N | - | N | - |
| Schultz 2014 | Schultz 2016 | N | - | N | - | N | - | Y | F |
| Apóstolo 2016 | Apóstolo 2017 | N | - | N | - | N | - | N | - |
| Goldstein 2016 | Goldstein 2017 | Y^a^ | T | Y^a^ | T | Y^a^ | T | Y^a^ | T |
|  | Shepherd-Banigan 2017 | Y | F | N | - | N | - | N | - |
|  | Befus 2018 | Y | F | N | - | N | - | N | - |
| Thomson 2016 | Thomson 2018 | Y | F | NR | - | Y | T | Y | F |
| Naik 2017 | Naik 2019 | N | - | NR | - | Y | F | Y | F |
| Melka 2018 | Melka 2020 | N | - | Y | F | N | - | Y | F |
| Petrovskaya 2019 | Antonio 2020 | N | - | N | - | N | - | Y | F |
| Santos 2019 | Santos 2019 | N | - | N | - | N | - | N | - |
| Skelton 2019 | Skelton 2020 | Y | F | Y | F | N | - | Y | F |
| Zhao 2020 | Zhao 2020 | N | - | NR | - | N | - | N | - |
| Lindekilde 2020 | Lindekilde 2021(a) | N | - | Y^b^ | F | N | - | Y^b^ | F |
| Griswold 2021 | Griswold 2021 | N | - | Y | F | Y | F | N | - |
| Hines 2018 | Khalil 2020 | N | - | N | - | N | - | N | - |
| Assi 2020 | Assi 2021 | N | - | N | - | N | - | N | - |
| Marano 2020 | Marano 2021 | N | - | N | - | N | - | N | - |
| van Esch 2016 | van Esch 2021 | Y | F | N | - | N | - | Y | F |
| Besnier 2019 | Besnier 2021 | N | - | N | - | N | - | N | - |
| Avşar 2018 | Avşar 2021 | N | - | N | - | N | - | Y^a^ | T |
| O'Malley 2020 | O'Malley 2021 | N | - | N | - | N | - | N | - |
| Lindekilde 2019 | Lindekilde 2021(b) | N | - | N | - | N | - | N | - |
| Griebeler 2012 | Griebeler 2014 | N | - | N | - | N | - | N | - |
| Alexandre 2017 | Alexandre 2021 | Y | F | N | - | N | - | N | - |
| Lugo 2017 | Lin 2019 | Y^a^ | T | Y^a^ | T | Y^a^ | T | Y^a^ | T |

Abbreviations: Y, both the protocol and its UR described the methodology and the inconsistencies arose between them; Y^a^, the publication of UR did not describe the methodology, but the protocol did; Y^b^, the publication of UR described the methodology, but its protocol did not; T, the deviations from the protocol were indicated in its publication; F, the deviations from the protocol were not indicated in its publication; NR, neither the protocol nor its publication described the methodology.

**Table S8.** Details of inconsistencies in the statistical analysis of the included URs.

| Umbrella review | | Overlap | | Certainty of evidence | | Summary of finding | | Data analysis | | Effect size | | Other* | |
| --- | --- | --- | --- | --- | --- | --- | --- | --- | --- | --- | --- | --- | --- |
| Protocol | Publication | Inconsistency | Transparency | Inconsistency | Transparency | Inconsistency | Transparency | Inconsistency | Transparency | Inconsistency | Transparency | Inconsistency | Transparency |
| Damery 2015 | Damery 2016 | NR | - | NR | - | Y | F | Y | F | NR | - | Y^a,^ ^1^ | F |
| Elliott 2017 | Elliott 2019 | Y^a^ | F | NR | - | Y | F | N | - | Y^b^ | F | N^1^ | - |
| Campbell 2014 | Campbell 2016 (a) | N | - | Y^a^ | F | Y | F | N | - | NR | - | NR | - |
|  | Campbell 2016 (b) | N | - | Y^a^ | F | Y | F | N | - | Y^b^ | F | NR | - |
|  | Campbell 2017 | N | - | Y^a^ | F | Y | F | N | - | Y^b^ | F | NR | - |
| Chai 2016 | Chai 2019 | N | - | N | - | Y | F | N | - | NR | - | NR | - |
| Jadczak 2016 | Jadczak 2018 | NR | - | Y^a^ | T | Y | T | N | - | NR | - | N^2^ | - |
| Qin 2019 | Chen 2020 | NR | - | N | - | NR | - | N | - | N | - | Y^1, 3^ | F |
| Tardif 2019 | Moore 2020 | NR | - | Y | T | Y | F |  | - | NR | - | NR | - |
| Dinsdale 2016 | Heslehurst 2020 | N | - | Y^a^ | F | Y | T | N | - | NR | - | Y^a,^ ^1^ | T |
| Schultz 2014 | Schultz 2016 | NR | - | N | - | N | - | Y | T | NR | - | Y^1^+N^2^ | T |
| Apóstolo 2016 | Apóstolo 2017 | N | - | NR | - | N | - | Y | T | NR | - | NR | - |
| Goldstein 2016 | Goldstein 2017 | NR | - | N | - | N | - | N | - | Y^b^ | F | Y^1^ | F |
|  | Shepherd-Banigan 2017 | NR | - | N | - | N | - | N | - | Y^b^ | F | N^1^ | - |
|  | Befus 2018 | NR | - | N | - | N | - | N | - | Y^b^ | F | N^1^ | - |
| Thomson 2016 | Thomson 2018 | NR | - | Y^a^ | F | NR | - | N | - | NR | - | Y^a,^ ^1^ | F |
| Naik 2017 | Naik 2019 | NR | - | NR | - | NR | - | N | - | NR | - | NR | - |
| Melka 2018 | Melka 2020 | N | - | Y^a^ | F | N | - | N | - | NR | - | NR | - |
| Petrovskaya 2019 | Antonio 2020 | N | - | N | - | Y | F | Y | T | NR | - | NR | - |
| Santos 2019 | Santos 2019 | N | - | N | - | N | - | N | - | NR | - | N^2^ |  |
| Skelton 2019 | Skelton 2020 | N | - | Y^a^ | F | N | - | Y | F | NR | - | Y^a,^ ^1^ | F |
| Zhao 2020 | Zhao 2020 | NR | - | NR | - | NR | - | N | - | NR | - | N^1, 3^ |  |
| Lindekilde 2020 | Lindekilde 2021(a) | N | - | NR | - | NR | - | N | - | NR | - | Y^3^ | T |
| Griswold 2021 | Griswold 2021 | NR | - | N | - | NR | - | N | - | N | - | Y^a,^ ^1^ | F |
| Hines 2018 | Khalil 2020 | NR | - | Y^a^ | F | N | - | N | - | NR | - | NR | - |
| Assi 2020 | Assi 2021 | N | - | NR | - | Y | T | N | - | NR | - | NR | - |
| Marano 2020 | Marano 2021 | Y^a^ | F | Y^b^ | F | N | - | Y | F | Y^b^ | F | Y^b,^ ^3^ | F |
| van Esch 2016 | van Esch 2021 | N | - | N | - | N | - | N | - | Y | F | N^1, 2, 3^ | - |
| Besnier 2019 | Besnier 2021 | N | - | NR | - | Y | F | N | - | NR | - | NR | - |
| Avşar 2018 | Avşar 2021 | NR | - | Y^b^ | F | NR | - | N | - | NR | - | Y^1^ | F |
| O'Malley 2020 | O'Malley 2021 | N | - | N | - | N | - | N | - | Y | T | Y^1^+N^2^ | T |
| Lindekilde 2019 | Lindekilde 2021(b) | NR | - | NR | - | NR | - | Y^b^ | F | NR | - | NR | - |
| Griebeler 2012 | Griebeler 2014 | NR | - | Y^a^ | F | N | - | Y | F | Y | T | Y^1, 2, 3^ | T |
| Alexandre 2017 | Alexandre 2021 | Y | F | NR | - | N | - | Y | T | Y^b^ | F | Y^b,^ ^1^ | F |
| Lugo 2017 | Lin 2019 | NR | - | NR | - | NR | - | N | - | NR | - | NR | - |

Abbreviations: Y, both the protocol and its UR described the methodology and the inconsistencies arose between them; Y^a^, the publication of UR did not describe the methodology, but the protocol did; Y^b^, the publication of UR described the methodology, but its protocol did not; T, the deviations from the protocol were indicated in its publication; F, the deviations from the protocol were not indicated in its publication; NR, neither the protocol nor its publication described the methodology; other*, including subgroup analysis (1), sensitivity analysis (2), and publication bias or small-study effects (3).

**Table S9.** Details of the frequency and transparency of inconsistencies in the included URs.

| Umbrella review | | Search strategy | | Inclusion Criteria | | Methods for screening | | Methods for data extraction | | Methodologicalquality assessment | | Statistical analysis | | Overall | |
| --- | --- | --- | --- | --- | --- | --- | --- | --- | --- | --- | --- | --- | --- | --- | --- |
| Protocol | Publication | Frequency | Transparency | Frequency | Transparency | Frequency | Transparency | Frequency | Transparency | Frequency | Transparency | Frequency | Transparency | Frequency | Transparency |
| Damery 2015 | Damery 2016 | 1 | 0 | 5 | 1 | 1 | 0 | 1 | 0 | 1 | 0 | 3 | 0 | 12 | 1 |
| Elliott 2017 | Elliott 2019 | 1 | 1 | 3 | 2 | 0 | 0 | 0 | 0 | 1 | 1 | 3 | 0 | 8 | 4 |
| Campbell 2014 | Campbell 2016 (a) | 2 | 1 | 3 | 0 | 0 | 0 | 0 | 0 | 0 | 0 | 2 | 0 | 7 | 1 |
|  | Campbell 2016 (b) | 2 | 1 | 3 | 0 | 0 | 0 | 0 | 0 | 0 | 0 | 3 | 0 | 8 | 1 |
|  | Campbell 2017 | 2 | 1 | 2 | 0 | 0 | 0 | 0 | 0 | 0 | 0 | 3 | 0 | 7 | 1 |
| Chai 2016 | Chai 2019 | 1 | 0 | 2 | 0 | 0 | 0 | 0 | 0 | 1 | 0 | 1 | 0 | 5 | 0 |
| Jadczak 2016 | Jadczak 2018 | 1 | 0 | 1 | 0 | 0 | 0 | 0 | 0 | 0 | 0 | 2 | 2 | 4 | 2 |
| Qin 2019 | Chen 2020 | 2 | 0 | 3 | 0 | 1 | 0 | 1 | 0 | 2 | 0 | 1 | 0 | 10 | 0 |
| Tardif 2019 | Moore 2020 | 1 | 0 | 4 | 0 | 1 | 0 | 1 | 0 | 0 | 0 | 2 | 1 | 9 | 1 |
| Dinsdale 2016 | Heslehurst 2020 | 2 | 0 | 3 | 2 | 1 | 0 | 0 | 0 | 1 | 0 | 3 | 2 | 10 | 4 |
| Schultz 2014 | Schultz 2016 | 0 | 0 | 3 | 1 | 0 | 0 | 1 | 0 | 0 | 0 | 2 | 2 | 6 | 3 |
| Apóstolo 2016 | Apóstolo 2017 | 0 | 0 | 2 | 2 | 0 | 0 | 0 | 0 | 0 | 0 | 1 | 1 | 3 | 3 |
| Goldstein 2016 | Goldstein 2017 | 2 | 1 | 3 | 0 | 1 | 1 | 1 | 1 | 2 | 2 | 2 | 0 | 11 | 5 |
|  | Shepherd-Banigan 2017 | 1 | 0 | 0 | 0 | 1 | 0 | 0 | 0 | 0 | 0 | 1 | 0 | 3 | 0 |
|  | Befus 2018 | 1 | 0 | 1 | 0 | 1 | 0 | 0 | 0 | 0 | 0 | 1 | 0 | 4 | 0 |
| Thomson 2016 | Thomson 2018 | 1 | 0 | 3 | 1 | 1 | 0 | 1 | 0 | 1 | 1 | 2 | 0 | 9 | 2 |
| Naik 2017 | Naik 2019 | 1 | 1 | 2 | 1 | 0 | 0 | 1 | 0 | 1 | 0 | 0 | 0 | 5 | 2 |
| Melka 2018 | Melka 2020 | 2 | 0 | 1 | 0 | 0 | 0 | 1 | 0 | 1 | 0 | 1 | 0 | 6 | 0 |
| Petrovskaya 2019 | Antonio 2020 | 2 | 1 | 3 | 2 | 0 | 0 | 1 | 0 | 0 | 0 | 2 | 1 | 8 | 4 |
| Santos 2019 | Santos 2019 | 0 | 0 | 1 | 0 | 0 | 0 | 0 | 0 | 0 | 0 | 0 | 0 | 1 | 0 |
| Skelton 2019 | Skelton 2020 | 0 | 0 | 1 | 0 | 1 | 0 | 1 | 0 | 1 | 0 | 3 | 0 | 7 | 0 |
| Zhao 2020 | Zhao 2020 | 0 | 0 | 3 | 0 | 0 | 0 | 0 | 0 | 0 | 0 | 0 | 0 | 3 | 0 |
| Lindekilde 2020 | Lindekilde 2021(a) | 0 | 0 | 2 | 0 | 0 | 0 | 1 | 0 | 1 | 0 | 1 | 1 | 5 | 1 |
| Griswold 2021 | Griswold 2021 | 1 | 0 | 3 | 1 | 0 | 0 | 0 | 0 | 2 | 0 | 1 | 0 | 7 | 1 |
| Hines 2018 | Khalil 2020 | 0 | 0 | 2 | 0 | 0 | 0 | 0 | 0 | 0 | 0 | 1 | 0 | 3 | 0 |
| Assi 2020 | Assi 2021 | 2 | 1 | 4 | 0 | 0 | 0 | 0 | 0 | 0 | 0 | 1 | 1 | 7 | 2 |
| Marano 2020 | Marano 2021 | 2 | 2 | 5 | 5 | 0 | 0 | 0 | 0 | 0 | 0 | 5 | 0 | 12 | 7 |
| van Esch 2016 | van Esch 2021 | 1 | 0 | 0 | 0 | 1 | 0 | 1 | 0 | 0 | 0 | 1 | 0 | 4 | 0 |
| Besnier 2019 | Besnier 2021 | 2 | 2 | 1 | 0 | 0 | 0 | 0 | 0 | 0 | 0 | 1 | 0 | 4 | 2 |
| Avşar 2018 | Avşar 2021 | 0 | 0 | 4 | 4 | 0 | 0 | 1 | 1 | 0 | 0 | 2 | 0 | 7 | 5 |
| O'Malley 2020 | O'Malley 2021 | 1 | 1 | 1 | 0 | 0 | 0 | 0 | 0 | 0 | 0 | 2 | 2 | 4 | 3 |
| Lindekilde 2019 | Lindekilde 2021(b) | 0 | 0 | 0 | 0 | 0 | 0 | 0 | 0 | 0 | 0 | 1 | 0 | 1 | 0 |
| Griebeler 2012 | Griebeler 2014 | 1 | 1 | 4 | 1 | 0 | 0 | 0 | 0 | 0 | 0 | 4 | 2 | 9 | 4 |
| Alexandre 2017 | Alexandre 2021 | 2 | 1 | 0 | 0 | 1 | 0 | 0 | 0 | 0 | 0 | 4 | 1 | 7 | 2 |
| Lugo 2017 | Lin 2019 | 2 | 1 | 5 | 5 | 1 | 1 | 1 | 1 | 2 | 2 | 0 | 0 | 11 | 10 |
| Overall | | 39 | 16 | 83 | 28 | 12 | 2 | 14 | 3 | 17 | 6 | 62 | 16 | 227 | 71 |
| Median (Interquartile range) | | 1 (0-2) | 0 (0-1) | 3 (1-3) | 0 (0-1) | 0 (0-1) | 0 (0-0) | 0 (0-1) | 0 (0-0) | 0 (0-1) | 0 (0-0) | 2 (1-3) | 0 (0-1) | 7 (4-9) | 1 (0-3) |
